# Comorbidities according to airflow limitation severity: data from comprehensive health examination in Japan

**DOI:** 10.1186/s12199-017-0620-0

**Published:** 2017-03-20

**Authors:** Shota Masuda, Hisamitsu Omori, Ayumi Onoue, Xi Lu, Kenichi Kubota, Noritaka Higashi, Yasuhiro Ogata, Takahiko Katoh

**Affiliations:** 1 Department of Public Health, Faculty of Life Sciences, Kumamoto University, 1-1-1 Honjou, Chuo-ku, Kumamoto, 860-8556 Japan; 2 Department of Biomedical Laboratory Sciences, Faculty of Life Sciences, Kumamoto University, 4-24-1 Kuhonji, Chuo-ku, Kumamoto, 862-0976 Japan; 3 Japanese Red Cross Kumamoto Health Care Center, 2-1-1 Nagamineminami, Higashi-ku, Kumamoto, 861-8528 Japan

**Keywords:** COPD, Comorbidity, Airflow limitation, Lung function, Health checkup

## Abstract

**Objectives:**

The present study aimed to investigate the relationship between airflow limitation (AL) severity and comorbidities in comprehensive health examination.

**Methods:**

This cross-sectional study included 6661 men and 6044 women aged 40–89 who underwent a lung function test during medical checkups. AL was defined as forced expiratory volume in 1 s/forced vital capacity of < 0.7. Logistic regression analysis was used to assess the association between AL severity and the presence of comorbidities.

**Results:**

When compared with the normal lung function group, subjects with AL had a higher prevalence of lung cancer (odd ratio (OR) 9.88, 95% confidence interval (CI) 3.88–25.14) in men, hypertension (OR 1.63, 95% CI 1.26–2.10) in women, diabetes and hyperglycemia (OR 1.23, 95% CI 1.02–1.49 in men, OR 1.61, 95% CI 1.18–2.20 in women) in men and women after adjusting for potential confounders. In men, lung cancer and MetS (the Joint Interim Statement: JIS) were significantly associated with moderate-to-very severe AL after adjustment. In women, hypertension, diabetes and hyperglycemia, MetS (JIS), and MetS (the Japanese Committee of the Criteria for MetS: JCCMS) were significantly associated with mild AL after adjustment. Hypertension was significantly associated with moderate-to-very severe AL after adjustment in women.

**Conclusions:**

Significant relationships were found between AL severity and the presence of comorbid lung cancer in men, hypertension in women, diabetes and hyperglycemia, and MetS in men and women. Knowledge of comorbidities associated with AL should be widely publicized to raise the awareness of chronic obstructive pulmonary disease (COPD).

## Introduction

Chronic obstructive pulmonary disease (COPD), a common preventable and treatable disease, is characterized by persistent airflow limitation that is usually progressive and associated with an enhanced chronic inflammatory response in the airways and the lung to noxious particles or gases [1, 2]. Exacerbations and comorbidities contribute to the overall severity in individual patients [1, 2]. COPD is a leading cause of morbidity and mortality worldwide and results in an economic and social burden that is both substantial and increasing [1, 2]. The World Health Organization (WHO) reported that COPD is the 3rd leading cause of death in the world and is presently the 5th leading cause of death among high-income countries, with a rate of 31 deaths per 10,000 people [3]. Furthermore, the burden of COPD is still expected to continue increasing [3].

COPD frequently coexists with other conditions often known as comorbidities that may have a significant impact on prognosis [1, 2]. Most common comorbidities are cardiovascular disease, hypertension, metabolic syndrome and diabetes, osteoporosis, musculoskeletal disease, lung cancer, and anxiety and depression [2]. These comorbidities have a significant impact on health status, health care, hospital admission and eventually death in patients with COPD [1, 2, 4–7]. The prevalence of individual comorbidities varies widely between different studies.

There are limited data available regarding the severity of airflow limitation (AL) and comorbidities, especially among subjects undergoing medical health checkups, in Japan. Therefore, the aim of the present study was to determine the relationship between AL and the common, chronic comorbid conditions of cardiovascular disease, hypertension, metabolic syndrome and diabetes, osteoporosis, lung cancer, and anxiety and depression.

## Materials and methods

### Subjects

**Fig. 1:**
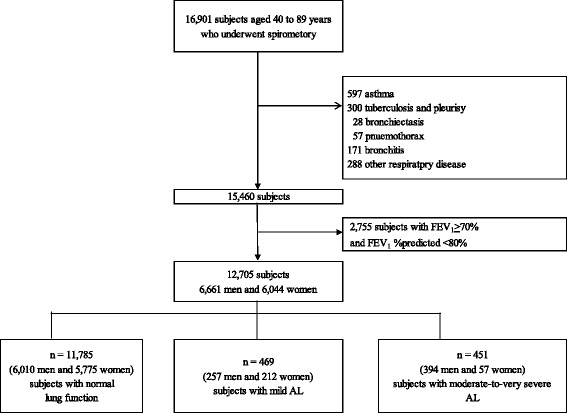
Flow chart for selecting the subjects with AL or normal lung function. *AL* airflow limitation, *FEV*_*1*_ forced expiratory volume in 1 s

Figure 1 shows the flow chart for selecting the subjects with either AL or a normal lung function. A total of 25,879 people visited the Japanese Red Cross Kumamoto Health Care Center for medical health checkups between April 2009 and March 2010. Of these, 16,901 subjects aged 40–89 years underwent comprehensive health examination that included spirometry, as previously described [8].

The comprehensive health examination included interview questionnaires, a physical examination, blood sampling, and spirometry, as previously described [8, 9]. The interview questionnaires were conducted by a trained public health nurse to obtain data regarding medical history including the use of medications, and smoking status. The never smokers consisted of those who denied any past or current smoking. The former smokers were those who reported smoking cessation prior to the examination. The current smokers were those who reported smoking at least one cigarette a day. Pack-years were calculated by multiplying the number of years of smoking by the average number of cigarettes smoked per day and dividing it by 20. All the participants were evaluated by a physician.

The subjects with asthma (number (n) = 597), tuberculosis and pleurisy (*n* = 300), bronchiectasis (*n* = 28), pneumothorax (*n* = 57), bronchitis (*n* = 171), and other respiratory diseases (*n* = 288) were excluded. In the present study, the subjects with lung cancer were included because lung cancer is an important comorbidity and a leading cause of death in COPD patients [1]. None of the subjects were diagnosed with COPD with acute exacerbation. The subjects with FEV_1/_FVC > 70% and %FEV_1_ < 80% (*n* = 2,755) were also excluded from this study. Data from a total of 12,705 subjects (6661 men and 6044 women) were including in the final analyses (Fig. 1, Table 1). None of the subjects had a history of exposure to workplace dust. Subjects were divided by lung function (11,785 subjects (6010 men and 5775 women)) had normal lung function, 469 subjects (257 men and 212 women) had mild AL, and 451 subjects (394 men and 57 women) had moderate-to-very severe AL) (Table 1).

**Table 1:** The characteristics of the study subjects based on lung function

Total (*n* = 12,705)	Normal (*n* = 11,785)		AL				*p* Value	
			Mild (*n* = 469)		Moderate-to-very severe (*n* = 451)			
	Men (*n* = 6,010)	Women (*n* = 5,775)	Men (*n* = 257)	Women (212)	Men (*n* = 394)	Women (*n* = 57)	Men	Women
Age, yr	56.4 (9.8)	55.1 (9.6)	63.0 (10.5)^**^	57.4 (10.6)^**^	59.7 (11.0)^**##^	55.4 (12.1)	<0.001	0.002
Height, cm	167.7 (6.2)	155.6 (5.7)	166.2 (6.5)^**^	155.6 (5.4)	167.8 (6.4)^##^	156.3 (7.0)	<0.001	0.626
Weight, kg	66.9 (9.7)	53.6 (8.4)	64.5 (8.8)^**^	54.2 (8.3)	66.3 (9.8)	52.7 (7.9)	<0.001	0.457
Abdominal circumference, cm	85.4 (7.8)	81.0 (8.9)	84.4 (6.9)	82.7 (7.8)^*^	85.7 (8.2)	80.5 (8.4)	0.122	0.023
BMI, kg/m^2^	23.7 (2.9)	22.1 (3.3)	23.3 (2.7)	22.4 (3.0)	23.5 (3.0)	21.6 (2.8)	0.03	0.243
Systolic blood pressure, mmHg	121.1 (16.8)	118.3 (17.8)	123.2 (18.9)	123.6 (18.4)^**^	125.8 (18.3)^**^	125.9 (18.0)^*^	<0.001	<0.001
Diastolic blood pressure, mmHg	76.2 (11.9)	72.8 (11.9)	75.1 (11.6)	75.4 (12.7)^*^	(74.8 (12.1)	72.0 (10.3)	0.032	0.006
Smoking status, n (%)								
Never smokers	1,995 (33.2)	5,216 (90.3)	71 (27.6)	192 (90.6)	(81 (20.5)	51 (89.5)		
Former smokers	2,298 (38.2)	257 (4.5)	(116 (45.1)	9 (4.2)	(165 (41.9)	4 (7.0)		
Current smokers	1,717 (28.6)	302 (5.2)	(70 (27.3)	11 (5.2)	148 (37.6)	2 (3.5)	<0.001	0.881
Pack-years	18.3 (20.1)	1.2 (4.7)	24.4 (24.4)^**^	1.9 (7.6)	28.6 (25.9)^**#^	1.3 (4.2)	<0.001	0.081
Lung function								
FVC, mL	4,029.0 (642.6)	3,280.0 (776.8)	4,227.6 (645.7)^**^	3,490.5 (787.2)^**^	3,242.4 (618.9)^**##^	2,306.0 (468.6)^**##^	<0.001	<0.001
FEV_1_, mL	3,226.1 (519.0)	2,645.3 (628.8)	2,789.2 (445.5)^**^	2,275.7 (506.7)^**^	2,023.2 (444.8)^**##^	1,427.0 (308.2)^**##^	<0.001	<0.001
FEV_1_/FVC, %	80.2 (5.2)	80.8 (5.3)	66.0 (3.3)^**^	65.4 (3.8)^**^	62.3 (6.8)^**##^	62.0 (6.7)^**##^	<0.001	<0.001
FEV_1_ % predicted, %	98.8 (14.0)	120.8 (29.2)	92.3 (11.7)^**^	105.7 (19.9)	63.8 (00312.4)^**##^	65.1 (12.1)^**##^	<0.001	<0.001
Laboratory data								
Fasting glucose, mg/dL	102.2 (19.5)	97.9 (17.5)	104.2 (19.2)	100.1 (18.7)	104.2 (22.3)	97.3 (15.3)	0.053	0.03
Triglycerides, mg/dL (interquartile range)	106.0 (74–157.0)	86.0 (63.0–127.0)	109.0 (82.5–162.5)	102.0 (71.0–141.8)	102.0 (75.0–147.0)	91.0 (68.5–115.5)	0.222	0.001
HDL cholesterol, mg/dL	61.7 (16.3)	68.7 (17.0)	61.6 (19.9)	66.9 (16.8)	61.5 (15.6)	73.2 (19.7)^#^	0.961	0.04
LDL cholesterol, mg/dL	119.3 (28.7)	119.2 (28.9)	116.8 (28.2)	119.6 (28.5)	116.7 (28.0)	111.7 (27.6)	0.09	0.15
White blood cell count,/μL	5,573.0 (1538.1)	5,227.3 (1454.6)	5,946.7 (1744.1)^*^	5,638.2 (1661.7)^**^	5,842.9 (1576.5)^*^	5,157.9 (1235.5)	<0.001	<0.001

Data are expressed as means (standard deviation), median (interquartile range), or as number (n) (percentage)

Airflow limitation (AL) was difined as FEV_1_/FVC < 0.7

Pack years = (number of cigareetes smoked per day × number of year smoked)/20

*BMI* body mass index, *FVC* forced vital capacity, *FEV*_*1*_ forced expiratory volume in one second, *HDL* high-density lipoprotein, *LDL* low-density lipoprotein, *hsCRP* hypersensitivity C-reactive protein

^*^ *p* < 0.05 compared with normal lung function

^#^ *p* < 0.05 compared with mild airflow limitation

All study subjects gave their informed consent to undergo a screening examination. Our research protocol was approved by the Human Ethics Committee of Kumamoto University (Numbers 84) and the Japanese Red Cross Kumamoto Health Care Center.

### Lung function tests

Spirometry was performed with an electronic spirometer (DISCOM-21 FX: CHEST MI, Tokyo, Japan) as previously described [8–13], using equipment and quality criteria that complied with international recommendations [14]. Reversibility tests were not performed for this study, and the classifications were based on pre-bronchodilator levels. According to Global Initiative for Chronic Obstructive Pulmonary Disease (GOLD) guidelines, we defined AL as an FEV_1_/FVC ratio of < 70% [1]. The predicted values were determined from the prediction equations published by the Japanese Respiratory Society (JRS) [15]: men, 0.036 × height (cm)-0.028 × age-1.178; women, 0.022 × height (cm)-0.022 × age-0.005. The criteria used for the AL staging were also developed according to GOLD guidelines, as follows: Stage I (mild AL): FEV_1_/FVC < 70% and %FEV_1_ > 80%; Stage II (moderate AL): FEV_1_/FVC < 70% and 50% < %FEV_1_ < 80%; Stage III (severe AL): FEV_1_/FVC < 70% and 30% < %FEV_1_ < 50%; and Stage IV (very severe AL): FEV_1_/FVC < 70% and %FEV_1_ < 30%. The subjects were divided into three groups: a control group (normal lung function), GOLD Stage I (mild AL), and GOLD Stages II–IV (moderate-to-very severe AL). The subjects with normal lung function were defined as having a FEV_1_/FVC > 70% and %FEV_1_ > 80%.

### Laboratory measurements

Following an overnight fast, blood samples were obtained to measure the serum levels of routine medical checkup indicators, including triglycerides, high-density lipoprotein cholesterol (HDL-C), low-density lipoprotein cholesterol (LDL-C), fasting glucose, and white blood cell count, as previously described [8].

### Comorbidities

The following comorbidities were evaluated according to the severity of AL in this study: lung cancer, hypertension, diabetes mellitus and hyperglycemia, dyslipidemia, metabolic syndrome, ischemic heart disease, osteoporosis, and depression and mental disease.

We ascertained the presence of lung cancer, ischemic heart disease, osteoporosis, and depression and mental disease by means of an interview. The presence of each comorbidity was confirmed by a physician. We defined hypertension as antihypertensive medication use, a systolic blood pressure of 130 mmHg or more, or a diastolic blood pressure of 85 mmHg or more. Dyslipidemia was defined as medication use, a triglyceride level of 150 mg/dL or more, an LDL cholesterol level of 140 mg/dL or more, or an HDL cholesterol level of less than 40 mg/dL, as described previously [8]. Diabetes and hyperglycemia were defined as medication use or a fasting glucose level of 110 mg/dL or more. We defined the presence of metabolic syndrome (MetS) using the following two criteria: the Joint Interim Statement (JIS) [16] and the Japanese Committee of the Criteria for MetS (JCCMS) [17]. MetS was diagnosed according to the JIS [16] when three or more of following components were present: 1) central obesity (waist circumference ≥ 90 cm in men and ≥ 80 cm in women); 2) high blood pressure (systolic blood pressure ≥ 130 mmHg and/or diastolic blood pressure ≥ 85 mmHg and/or current use of medication for hypertension); 3) high triglyceride (triglyceride > 150 mg/dL and/or current use of medication for triglyceride); 4) low HDL cholesterol (HDL cholesterol < 40 mg/dL in men or < 50 mg/dL in women and/or current use of medication for HDL cholesterol); and 5) impaired fasting glucose (fasting glucose ≥ 100 mg/dL and/or current use of medication for diabetes mellitus).

Furthermore, we diagnosed metabolic syndrome according to Japanese diagnostic criteria [17]: a waist circumference of at least 85 cm for men and 90 cm for women, and 2 or more of the following components: 1) a triglyceride level of 150 mg/dL and/or current use of medication for triglyceride or more and/or an HDL cholesterol level of less than 40 mg/dL and/or current use of medication for HDL cholesterol; 2) a systolic blood pressure of 130 mmHg or more and/or a diastolic blood pressure of 85 mmHg or more and/or current use of medication for hypertension; and 3) a fasting glucose of 110 mg/dL or more and/or current use of medication for diabetes mellitus.

### Statistical analyses

Data were presented as number of cases, mean (standard deviation), or median with interquartile range. Differences among normal lung function, mild AL, and moderate-to-very severe AL were compared using a one-way analysis of variance or Kruskal-Wallis test in case of not normally distributed data followed by Scheffe’s post-hoc tests for continuous variables and a chi-squared test for categorical variables. A multivariate logistic regression model adjusted for age, BMI, and smoking was used to assess the relationship between severity of AL and comorbidities, with “normal lung function” as the reference. All statistical analyses were performed with IBM SPSS Statistics 22.0 software. Whether the data showed normal distribution was assessed by Shapiro-Wilk test. Values of *p* < 0.05 were considered to be statistically significant. There are no missing data in the present study.

## Results

### Study population characteristics

Table 1 shows the characteristics of the study subjects based on their lung function status. The prevalence of AL in this study population for the GOLD stages “mild” and “moderate-to-very severe” AL were 3.7% (*n* = 469) and 3.5% (*n* = 451), respectively. Overall prevalence of AL was 7.2%. In men, 3.9% (*n* = 257) had mild airflow limitation and 5.9% (*n* = 394) had moderate-to-very severe airflow limitation. In women, 3.5% (*n* = 212) had mild airflow limitation and 1.0% (*n* = 57) had moderate-to-very severe airflow limitation. Among 394 men with moderate-to-very severe AL, most of the subjects were at moderate (*n* = 343, 5.15%) compared to severe (*n* = 45, 0.68%) and very severe (*n* = 6, 0.09%). In 57 women with moderate-to-very severe AL, prevalence of the subjects with moderate, severe and very severe AL were 0.83% (*n* = 50), 0.12% (*n* = 7), and 0% (*n* = 0), respectively.

In this study, 6.6% (*n* = 31) of subjects with mild AL and 1.6% (*n* = 7) of subjects with moderate-to-very severe AL had been diagnosed with COPD/emphysema. Remaining had not been diagnosed with COPD/emphysema.

Significant differences in the lung function status were found with regard to age in men and women, height, weight, smoking status, pack-years in men, abdominal circumference, fasting glucose, triglyceride, and HDL-cholesterol in women. White blood cell count was significantly higher in the subjects with mild and moderate-to-very severe AL than in those with normal lung function in men.

### Comorbidities

Table 2 shows the prevalence of comorbidities between subjects with mild AL, moderate-to-very severe AL, and normal lung function. Hypertension was the most prevalent comorbidity in men and women in the subjects with AL. The present study shows a low ratio of ischemic heart disease, osteoporosis, and depression and mental disease. When compared with the normal lung function group, subjects in AL group had a significantly higher prevalence of lung cancer and diabetes and hyperglycemia in men and lung cancer, hypertension, diabetes and hyperglycemia and osteoporosis in women. No significant difference were found in dyslipidemia, Mets (JIS), Mets (JCCMS), ischemic heart disease, osteoporosis, and depression and mental disease in the different groups of AL severity.

**Table 2:** Prevalence of comorbidites between subjects with mild AL, moderate-to-severe AL, and normal lung function

Comorbidity	Normal *n* = 11,785	AL		*p* value
		Mild *n* = 469	Moderate-to-very severe *n* = 451	
Men (*n* = 6,661)	*n* = 6,010	*n* = 257	*n* = 394	
Lumg cancer	9 (0.15)	3 (1.17)	7 (1.78)	<0.001
Hypertension	2,602 (43.29)	127 (49.42)	210 (53.30)	0.064
Diabetes and hyperglycemia	1,241 (20.65)	65 (25.29)	102 (22.62)	0.012
Dyslipidemia	2,887 (48.04)	118 (45.91)	182 (46.19)	0.637
MetS (JIS)	1,009 (16.79)	44 (17.12)	84 (21.32)	0.068
MetS (JCCMS)	964 (16.04)	46 (18.11)	68 (17.26)	0.611
Ischemic heart disease	164 (2.73)	7 (2.72)	14 (3.55)	0.627
Osteoporosis	8 (0.13)	0 (0)	1 (0.25)	0.684
Depression and mental disease	17 (0.28)	0 (0)	1 (0.25)	0.692
Women (*n* = 6,044)	*n* = 5,775	*n* = 212	*n* = 57	
Lumg cancer	15 (0.26)	2 (0.94)	1 (1.75)	0.026
Hypertension	1,886 (32.66)	97 (45.75)	25 (43.86)	<0.001
Diabetes and hyperglycemia	754 (13.06)	44 (20.75)	9 (15.79)	0.005
Dyslipidemia	2,433 (42.13)	94 (44.34)	17 (29.82)	0.138
MetS (JIS)	1,024 (17.73)	51 (24.06)	9 (15.79)	0.057
MetS (JCCMS)	200 (3.46)	14 (6.60)	2 (3.51)	0.054
Ischemic heart disease	61 (1.06)	3 (1.42)	0 (0)	0.648
Osteoporosis	316 (5.47)	19 (8.96)	6 (10.53)	0.027
Depression and mental disease	15 (0.26)	1 (0.47)	0 (0)	0.779

Data presented are number (percent)

Ischemic heart disease; including angina pectoris and myocardial infarction Airflow limitation (AL) was dfined as FEV_1_/FVC < 0.7

Hypertension; antihypertensive medication use or systolic blood pressure ≧ 130 mmHg or diastolc blood pressure ≧ 85 mmHg Diabetes and hypergfycemia; blood glucose-lowerning medication use or elevated fasting glucose ≧ 110 Dyslipidemia; medication use or triglycerides ≧ 150 mg/dl, HDL-C < 40 mg/dl or LDL-C ≧ 140 mg/dl

*AL* airflow limitation, *MetS* metabolic syndrome, *JIS* Joint Interim Statement, *JCCMS* Japanese Committee of Criteria for MetS

Table 3 displays the relationship between AL and comorbidities according to lung function. In logistic regression models adjusting for sex, age, BMI, and smoking status, the risks of lung cancer (odd ratio (OR): 9.88; 95% confidence interval (CI): 3.88–25.14), and diabetes and hyperglycemia (OR: 1.23; 95% CI: 1.02–1.49) were higher in subjects with AL compared to those with normal lung function and in logistic regression models adjusting for age and smoking status, the risk of MetS (JIS) (OR: 1.32; 95% CI: 1.02–1.69) was higher in subjects with moderate-to-very severe airflow limitation in men. However, hypertension, dyslipidemia, MetS (JCCMS) and ischemic heart diseases were not significantly associated with AL in men in the present study.

**Table 3:** Relationship between AL and comorbidities according to lung function

Comorbidity	Normal *n* = 11,785	AL total *n* = 920	*p* Value	AL			
				Mild *n* = 469	*p* Value	Moderate-to-very severe *n* = 451	*p* Value
Men (*n* = 6,661)	*n* = 6,010	*n* = 651		*n* = 257		*n* = 394	
Lung cancer							
Crude OR (95% CI)	1.00	10.40 (4.21–25.69)	<0.001	7.88 (2.12–29.27)	0.002	12.06 (4.47–32.56)	<0.001
Adjusted OR (95% CI)	1.00	9.88 (3.88–25.14)	<0.001	6.52 (1.70–24.99)	0.006	12.58 (4.53–34.94)	<0.001
Hypertension							
Crude OR (95% CI)	1.00	1.21 (1.03–1.42)	0.024	1.28 (1.00–1.64)	0.053	1.16 (0.95–1.42)	0.156
Adjusted OR (95% CI)	1.00	1.08 (0.91–1.27)	0.404	1.08 (0.83–1.39)	0.585	1.08 (0.87–1.33)	0.506
Diabetes and hyperglycemia							
Crude OR (95% CI)	1.00	1.33 (1.10–1.60)	0.003	1.30 (0.98–1.74)	0.073	1.34 (1.06–1.70)	0.014
Adjusted OR (95% CI)	1.00	1.23 (1.02–1.49)	0.034	1.19 (0.89–1.60)	0.245	1.26 (0.99–1.60)	0.06
Dyslipidemia							
Crude OR (95% CI)	1.00	0.93 (0.79–1.09)	0.343	0.92 (0.72–1.18)	0.505	0.93 (0.76–1.14)	0.478
Adjusted OR (95% CI)	1.00	0.95 (0.81–1.13)	0.576	0.98 (0.76–1.26)	0.862	0.94 (0.76–1.16)	0.551
MetS (JIS)							
Crude OR (95% CI)	1.00	1.21 (0.99–1.49)	0.065	1.02 (0.74–1.43)	0.889	1.34 (1.05–1.73)	0.021
Adjusted^*^ OR (95% CI)	1.00	1.21 (0.98–1.49)	0.075	1.05 (0.75–1.46)	0.797	1.32 (1.02–1.69)	0.034
MetS (JCCMS)							
Crude OR (95% CI)	1.00	1.11 (0.90–1.38)	0.333	1.14 (0.82–1.58)	0.428	1.09 (0.83–1.43)	0.524
Adjusted^*^ OR (95% CI	1.00	1.05 (0.85–1.31)	0.648	1.08 (0.77–1.50)	0.665	1.04 (0.79–1.36)	0.797
Ischemic heart disease							
Crude OR (95% CI)	1.00	1.12 (0.75–1.89)	0.464	1.00 (0.46–2.15)	0.996	1.31 (0.75–2.29)	0.336
Adjusted OR (95% CI)	1.00	0.86 (0.53–1.38)	0.524	0.65 (0.30–1.41)	0.27	1.02 (0.58–1.81)	0.937
Women (n–6,044)	*n* = 5,775	*n* = 269		*n* = 212		*n* = 57	
Lung cancer							
Crude OR (95% CI)	1.00	4.33 (1.25–15.05)	0.02	3.66 (0.83–16.10)	0.086	6.86 (0.89–52.80)	0.065
Adjusted OR (95% CI)	1.00	3.42 (0.97–12.11)	0.057	2.82 (0.63–12.64)	0.176	5.95 (0.75–47.32)	0.09
Hypertension							
Crude OR (95% CI)	1.00	1.71 (1.34–2.19)	<0.001	1.74 (1.32–2.29)	<0.001	1.61 (0.95–2.73)	0.08
Adjusted OR (95% CI)	1.00	1.63 (1.26–2.10)	<0.001	1.59 (1.20–2.11)	0.001	1.79 (1.04–3.10)	0.037
Diabetes and hyperglycemia							
Crude OR (95% CI)	1.00	1.63 (1.20–2.23)	0.002	1.74 (1.24–2.45)	0.001	1.25 (0.61–2.56)	0.54
Adjusted OR (95% CI)	1.00	1.61 (1.18–2.20)	0.003	1.68 (1.19–2.37)	0.003	1.34 (0.65–2.75)	0.43
Dyslipidemia							
Crude OR (95% CI)	1.00	0.97 (0.75–1.24)	0.779	1.09 (0.83–1.44)	0.522	0.58 (0.33–1.78)	0.70
Adjusted OR (95% CI)	1.00	0.90 (0.70–1.16)	0.418	1.00 (0.75–1.32)	0.982	0.89 (0.43–1.82)	0.74
MetS (JIS)							
Crude OR (95% CI)	1.00	1.33 (0.99–1.79)	0.057	1.47 (1.07–2.03)	0.019	0.87 (0.43–1.78)	0.70
Adjusted^*^ OR (95% CI)	1.00	1.31 (0.97–1.76)	0.075	1.43 (1.04–1.98)	0.03	0.89 (0.43–1.82)	0.74
MetS (JCCMS)							
Crude OR (95% CI)	1.00	1.76 (1.04–2.98)	0.034	1.97 (1.13–3.45)	0.018	1.01 (0.25–4.19)	0.99
Adjusted^*^ OR (95% CI)	1.00	1.64 (0.97–2.78)	0.067	1.83 (1.04–3.21)	0.036	0.94 (0.23–3.93)	0.94
Osteoporosis							
Crude OR (95% CI)	1.00	1.77 (1.16–2.71)	0.009	1.70 (1.05–2.76)	0.032	2.03 (0.87–4.77)	0.10
Adjusted OR (95% CI)	1.00	1.33 (0.83–2.14)	0.243	1.24 (0.73–2.12)	0.428	1.73 (0.64–4.68)	0.28

Adjusted for age, body mass index, smoking status, ^*^ adjusted for age and smoking status

Airflow limitation (AL) was difined as FEV_1_/FVC < 0.7

Hypertension; antihypertensive medication use or systolic blood pressure ≧130 mmHg or diastolic blood pressure ≧85 mmHg Diabetes and hyperglycemia; blood glucose-lowerning medication use or elevated fasting glucose ≧110 Dyslipidemia; medication use or triglycerides ≧150 mg/dl, HDL-C < 40 mg/dl or LDL-C≧140 mg/dl MetS (JCCMS) Dyslipidemia

*AL* airflow limitation, *MetS* metabolic syndrome, *JIS* Joint Interim Statement, *JCCMS* Japanese Committee of Criteria for MetS, *OR* odds ratio, *CI* confidence interval

In women, the risks of hypertension were significantly higher in subjects with AL (OR: 1.63; 95% CI: 1.26–2.10), mild AL (OR: 1.59; 95% CI: 1.20–2.11), and moderate-to-very severe AL (OR: 1.79; 95% CI: 1.04–3.10) compared to those with normal lung function after adjusting for age, BMI, smoking status. The risks of diabetes and hyperglycemia were significantly higher in subjects with AL (OR: 1.61; 95% CI: 1.18–2.20) and mild AL (OR: 1.68; 95% CI: 1.19–2.37) compared to those with normal lung function after adjusting for age, BMI, smoking status. The risks of MetS (JIS) (OR: 1.43; 95% CI: 1.04–1.98) and MetS (JCCMS) (OR: 1.83; 95% CI: 1.04–3.21) were significantly higher in subjects with mild airflow limitation compared to those with normal lung function after adjusting for age and smoking status in women. However, the risks of lung cancer, dyslipidemia and osteoporosis were not significantly higher in subjects with AL after adjusting for age, BMI, smoking status in women in the present study.

## Discussion

This study focused on the prevalence and risks of comorbidities in those who received comprehensive health examination according to their lung function. The currently available data regarding the severity of AL and comorbidities, especially among subjects undergoing medical health checkups, are limited.

The main findings of this study are that the prevalence of lung cancer, diabetes and hyperglycemia, and MetS (JIS) were significantly higher in subjects with moderate-to-very severe AL. In men, the risks of lung cancer, diabetes and hyperglycemia, and MetS (JIS) were significantly higher in subjects with AL compared to those with normal lung function. In women, the risks of hypertension, diabetes and hyperglycemia, MetS (JIS) and MetS (JCCMS) were higher in subjects with AL compared to those with normal lung function.

The document of Global Initiative for Chronic Obstructive Pulmonary Disease (GOLD) reported that lung cancer is frequently seen in patients with COPD and has been found to be the most frequent cause of death in patients with mild to moderate COPD [1, 2]. In this study, we demonstrated that the odds ratio for having lung cancer compared to those with normal lung function was 6.52 in subjects with mild AL and 12.58 in subjects with moderate-to-very severe AL in men, whereas there was no significant difference in women. In this study, we could not obtain the information about the histologic type of lung cancer from interview questionnaires. Several longitudinal studies demonstrated the relationship between COPD and lung cancer [18–20]. Mannino et al. demonstrated a hazard ratio of 2.8 (adjusted for smoking, age, sex, race, and education) in patients with moderate to severe COPD and a risk proportional to the severity of the AL in an analysis of 22-years follow-up data [18]. De Torres et al. showed that mild and moderate stages of COPD are associated with a greater risk for developing lung cancer [19]. COPD has been identified as an independent risk factor for lung cancer [18–20]. In the present cross-sectional study, we observed an association between airflow limitation severity and lung cancer in men. As with any cross-sectional study, there may be a potential reverse causation between airflow limitation and lung cancer.

Our findings could have implications in the management of subjects with AL encountered in the setting of medical health checkups and in lung cancer screening program.

The exact reason why some smokers develop COPD, some develop cancer and some develop both diseases is not known [21]. The presence of chronic smoldering inflammation has been postulated as the possible underlying mechanism linking cancer and COPD caused by cigarette smoke exposure [19, 21]. Recent studies have proposed chromosomal loci and/or candidate genes associated with lung function, COPD and lung cancer [21]. It would be very useful to further understand the overlapping pathways of COPD and lung cancer [21].

Sex differences have raised some controversy in the literature regarding COPD and lung cancer [21]. Loganathan et al. showed a significantly lower prevalence of COPD in females diagnosed with lung cancer than in males [22]. Our findings are in line with the previous study. They suggested that sex-based differences should be taken into account when building up strategies for lung cancer screening. Larger epidemiological studies are needed in this area.

Hypertension is consistently one of the most prevalent comorbid diagnosis in COPD patients reported in 40–60% [5] and has implications for prognosis [1]. Mannino et al. reported a prevalence of hypertension of 34% in normal subjects, rising to 40% in GOLD stage I patients, 44% in GOLD stageIIand 51% in GOLD stage III and IV [5]. In the multivariate analysis, the odds ratio for having hypertension compared with normal subjects was 1.4 in GOLD stage I and 1.6 in GOLD stages II and IV [5]. In this study, hypertension (OR: 1.63; 95% CI: 1.26–2.10) is higher in subjects with AL compared to those with normal lung function in women, whereas there was no significant difference in men. Larger epidemiological studies are needed regarding gender-based difference.

The OR having diabetes and hyperglycemia compared with normal lung function was 1.23 for men and 1.61 for women in total AL, and 1.68 in mild AL for women. The pathophysiological link between COPD and diabetes is not entirely understood, although thought to involve systemic inflammation with central roles for IL-6 and TNF-α [23]. We found that the prevalence of MetS was higher when using JIS criteria than when using JCCMS criteria, especially in women, which were consistent with the study by Hu H. et al. [24]. Hu H et al. demonstrated that the JIS criteria can detect more people who later develop diabetes mellitus than does the JCCMS criteria [24]. In men, MetS (JIS) (OR, 1.32; 95% CI, 1.02–1.69) were significantly associated with moderate-to-very severe AL, after adjusting for sex, age, and smoking status. However, we found that MetS (JCCMS) was not significantly associated with moderate-to-very severe AL after adjusting for sex, age, and smoking status. In women, MetS (JIS) (OR, 1.68; 95% CI, 1.19–2.37) and MetS (JCCMS) (OR, 1.83; 95% CI, 1.04–3.21) were significantly associated with mild AL, after adjusting for sex, age, and smoking status. Previous studies have reported that diabetes and Mets are more frequent in COPD patients who are at the earlier stages of COPD [25–27]. The reason of the gender-difference is not clear. Further studies are needed to better understand the gender-difference. Our findings in men are consistent with our previous study [11]. These criteria differ in several aspects, including the cut-off points of waist circumference (WC), handling of the WC component (prerequisite or optional for the diagnosis of MetS), and the criteria of hyperglycemia and dyslipidemia [24]. These differences have led to confusion regarding the choice of the criteria to diagnose MetS [24]. Hu H. et al. very recently demonstrated that waist circumference cut-offs of 85 cm for men and 80 cm for women are appropriate in the Japanese population [24]. Additional research is needed to clarify this aspect of criteria.

Dyslipidemia is one of several parameters employed to diagnose metabolic syndrome [16, 17]. However, most studies have not demonstrated significant differences in the prevalence of dyslipidemia between COPD patients and control subjects [23, 28]. Our findings are consistent with these previous studies.

Compared with the previous study by Smith et al., our study demonstrated a low ratio of ischemic heart disease [29]. According to the document of Global Initiative for Chronic Obstructive Lung Disease (GOLD), cardiovascular disease is a major comorbidity in COPD and probably both the most frequent and most important [1, 2]. However, we found that ischemic heart disease is less prevalent in this study. In addition, ischemic heart disease was not significantly associated with AL after adjusting for sex, age, BMI, and smoking status in the present study. There are conflicting results regarding the association between COPD severity and the risk of cardiovascular comorbidity [25]. A review by Takahashi S et al. [25] demonstrated that the comorbidity spectrum of Japanese COPD patients seems to differ from that of Westerners. For example, they reported that cardiovascular disease is less prevalent Japanese COPD patients. The fact that the prevalence of cardiovascular comorbidities in Japanese COPD patients differs from that in Western patients may arise from ethnic or genetic differences, or from environmental differences including lifestyle and socioeconomic factors [25].

Studies have shown that osteoporosis, anxiety and depression are major comorbidities in COPD [1, 2, 29]. In this study, we observed a low ratio of osteoporosis, anxiety and depression. Further research is needed to elucidate the relationship between AL and these comorbidities.

COPD is characterized by persistent systemic inflammation [1, 6]. Previous research found that the group of patients with severe COPD had significantly higher circulating leukocyte [6]. Our results are in line with this study. Systemic inflammation has been widely reported to be a key link between COPD and some comorbidities [23]. The source of systemic inflammation in COPD may be the results of a spill-over of airway and lung parenchymal processes [23]. While the causal relationship between COPD and comorbidities is not entirely clear, inactivity and systemic inflammation have been suggested as part of the mechanism for the comorbidities associated with COPD and may relate to the natural course of the disease [30, 31].

The strength of this study is estimating the prevalence of AL and comorbidities among subjects undergoing medical checkup. In this study, the prevalence of diagnosed COPD/emphysema among subjects with AL was only 6.6% in mild AL and 1.6% in moderate-to-very severe AL. Remaining had not been diagnosed with any respiratory diseases, suggesting a high degree of under-diagnosis of COPD. Conclusively, earlier diagnosis and intervention of COPD and its comorbidities could improve the prognosis of COPD for individual patients and reduce the disease burden of COPD on the society.

There are several limitations associated with the present study. First, we did not employ reversibility testing, since our Institutional Review Board considered it unacceptable in the absence of a high suspicion of disease. For this reason, the subjects with AL may have included subjects with a post-bronchodilator FEV_1_/FVC ratio greater than 70%. Our study excluded any subjects who had ever received a diagnosis of asthma or other respiratory diseases except for lung cancer. Therefore, these individuals could possibly have COPD, and they may have even had asthma; thus, we expressed “AL” instead of COPD. This limitation has also been reported in the previous studies [8, 9]. A modified GOLD definition that omits bronchodilation has become widely adopted by population-based epidemiological studies [32]. Second, the presence of lung cancer, ischemic heart disease, osteoporosis, and depression and mental disease was confirmed by the interview by a trained public health nurse and a physician. The methodology used to report the presence of a comorbidity that is not completely objective because it was not based on confirmatory tests for each disease.

Third, because of the cross-sectional design, we cannot rule out the possibility that reverse causation may explain our results. Further larger-scale prospective studies are needed to further confirm our findings. Fourth, the present study was a single-center study performed with subjects who underwent a medical health checkup. This may limit applicability across different centers.

Despite these limitations, we consider the present study to be worthwhile because it is the first to reveal the relationship between AL severity and various comorbidities in comprehensive health examination, as far as the authors know.

## Conclusions

In conclusion, a significant relationship was found between AL and the presence of comorbid lung cancer in men, hypertension in women, diabetes and hyperglycemia, and MetS in men and women. Further research investigating gender-based difference of comorbidity is needed. Similarly, further research is required to fully understand the relationships and underlying mechanisms between airflow limitation and the comorbidities. Our findings could have implications in the management of subjects with AL on medical health checkups. Efforts aimed at the earlier detection of AL and the identification of comorbidities may become integral for the reduction of the disease burden of COPD on the society. Knowledge of comorbidities associated with AL should be widely publicized to raise the awareness of COPD.
